# The Variability and Determinants of Serotiny in *Pinus Yunnanensis*

**DOI:** 10.1002/ece3.72838

**Published:** 2026-01-05

**Authors:** Ying Liu, Haichuan Lin, Dongli Yu, Zihan Zhang, Wuchao Gao, Dongyu Cao, Dachuan Dai, Xinglei Cui

**Affiliations:** ^1^ College of Forestry Sichuan Agricultural University Chengdu China; ^2^ Sichuan Mt. Emei Forest Ecosystem National Observation and Research Station Emei China; ^3^ Forest Ecology and Conservation in the Upper Reaches of the Yangtze River Key Laboratory of Sichuan Province Chengdu China

**Keywords:** fire‐adaptive trait, physiological characteristic, *Pinus yunnanensis*, serotiny, wildfire

## Abstract

Serotiny, a key fire‐adaptive trait, enables certain plants to retain seeds in closed cones until triggered by fire, thus facilitating post‐fire regeneration. Although serotiny has been observed in many species, the physiological and environmental mechanisms that regulate this trait remain poorly understood. In this study, we investigated the variation in serotiny level within *Pinus yunnanensis*, a pine species native to fire‐prone landscapes in southwestern China. Serotiny level varied significantly among populations and was significantly associated with environmental factors and cone physiological characteristics, including Bio2 (mean diurnal temperature range), Bio5 (maximum temperature of the warmest month), soil pH, total soil phosphorus, and cone resin content. Cone resin content exhibited the strongest direct positive effect on serotiny. Soil pH and phosphorus concentration negatively affected resin content in cones, thus indirectly reducing serotiny level. Bio2 enhanced serotiny indirectly by acidifying soil and promoting resin synthesis, while Bio5 decreased serotiny indirectly by increasing phosphorus availability and limiting resin accumulation. Recent fire activity and fire radiative power did not appear to have a significant effect on serotiny in *P. yunnanensis* . These findings enhance our understanding of how serotiny evolves under the combined pressures of climate and soil conditions in fire‐adapted ecosystems.

## Graphical abstract

This study investigates the intraspecific variation in serotiny level among three varieties of Pinus yunnanensis. Using field data and structural equation modeling, we found that cone resin content is the key physiological driver of serotiny, indirectly shaped by climate and soil factors. Our findings highlight the dominant role of long‐term environmental interactions over fire activity in shaping this fire‐adaptive trait.

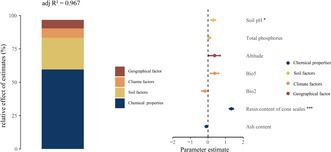

## 1 Introduction

Serotiny, the prolonged retention of seeds within cones after maturation, is a pivotal fire‐adaptive trait that enables the formation of a canopy seed bank (Gutterman and Ginott 1994; Lamont et al. 1991). Canopy seed banks offer a selective advantage in fire‐prone ecosystems by maintaining a reliable source of seeds for post‐fire regeneration, particularly when the interval between fires exceeds the time required for a species to reach reproductive maturity (Lamont and Enright 2000; Keeley et al. 2011). Serotiny can enhance both species persistence and ecosystem resilience in fire‐prone environments (Enright et al. 1998a; Lamont and Groom 2013). Serotiny has been documented in more than 1000 plant species across diverse families, including Cupressaceae, Casuarinaceae, and Pinaceae (Lamont 1991; Buma et al. 2013; Tada et al. 2024). This trait can be quantified by serotiny level, the proportion of mature cones retaining seeds relative to total cone production, which can exhibit substantial variability both among and within species (Schoennagel et al. 2003; Vincenzi and Piotti 2014).

Fire regime is widely recognized as the primary evolutionary driver of serotiny. For instance, populations of *Pinus halepensis* in regions with frequent wildfires tend to exhibit higher serotiny levels compared to those in areas with infrequent fire events (Romero and Ganteaume 2020). However, fire regime characteristics alone are insufficient to fully explain the observed variation in this trait. Other environmental factors, particularly temperature and aridity, also play crucial roles in shaping serotiny. For instance, in some species, such as *Pinus halepensis* and *Pinus contorta* , air moisture can trigger cone dehiscence even in the absence of fire (Hellum and Barker 1980; Nathan et al. 1999). Similarly, extremely high temperatures and arid conditions are also able to act as cues for cone opening (Talluto et al. 2017). These non‐fire‐induced mechanisms can lead to seed release, thereby reducing serotiny levels in affected populations.

Serotiny level is intricately linked to the chemical composition of cones, especially the resin content, which serves as a natural adhesive to maintain cone closure. Strong correlations were detected between resin content in cone scales and serotiny level across, as well as within species (Su et al. 2017; Wang et al. 2020), suggesting that higher resin concentrations reinforce serotinous cone structure. Resins are primarily composed of terpenoids and their synthesis is especially sensitive to temperature. Elevated temperatures can inhibit terpenoid biosynthesis (Loreto and Schnitzler 2010; Gershenzon 1994), and in warm environments, plants may reallocate resources from defense mechanisms to reproduction, further reducing resin production (Feeny 1976; Endara and Coley 2011). The physiological trade‐off compromises cone integrity and may lead to premature cone opening, thereby lowering serotiny level. However, the specific impact of climate conditions on resin synthesis and, consequently, on serotiny level has been relatively unexplored.

**FIGURE 1:**
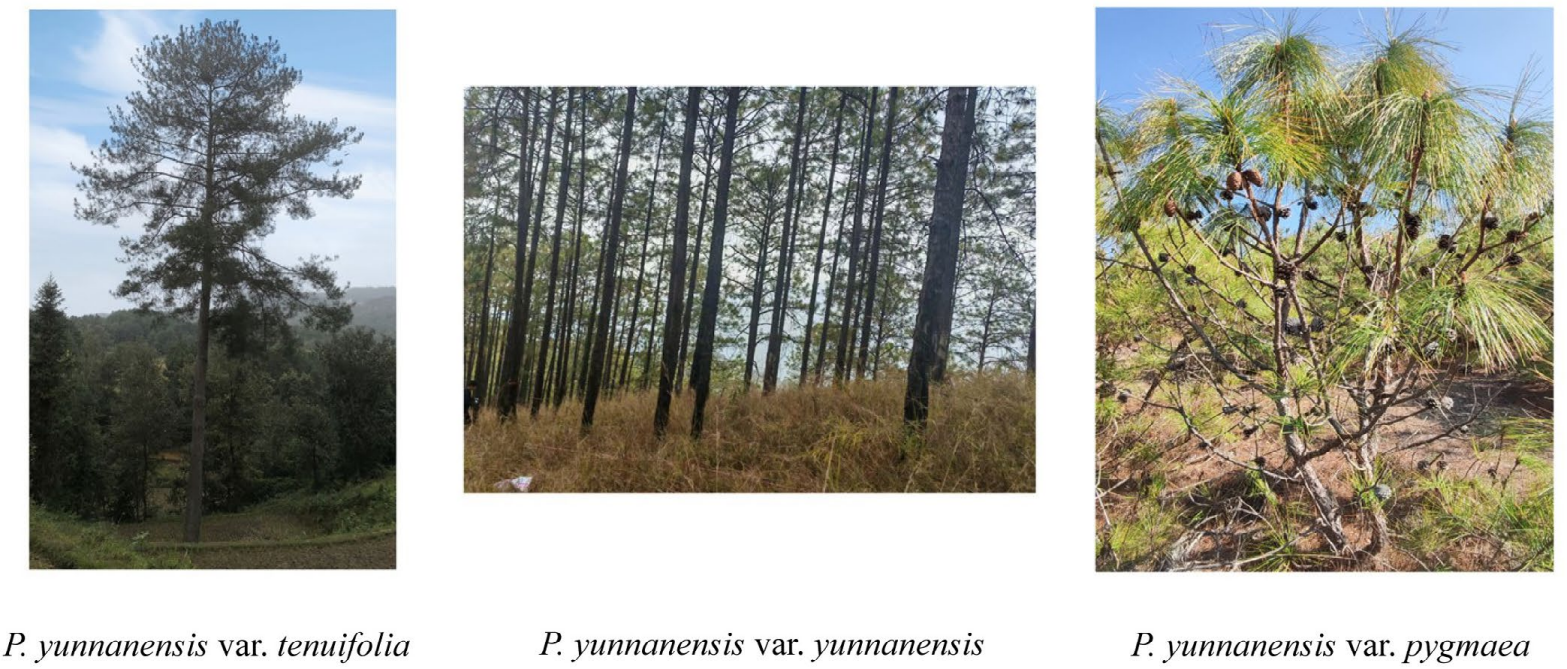
Morphological variation among the three recognized varieties of *Pinus yunnanensis*. From left to right: *P. yunnanensis* var. *tenuifolia*, *P. yunnanensis* var. *yunnanensis*, and *P. yunnanensis* var. *pygmaea*. These varieties differ markedly in tree height, crown structure, and habitat preference.

*Pinus yunnanensis* is an endemic conifer native to southwestern China, a region frequently experiencing wildfires (Shen et al. 2020). This species comprises three recognized varieties: *P. yunnanensis* var. *yunnanensis*, var. *tenuifolia*, and var. *pygmaea*, which differ markedly in morphology and occupy varied habitats. Var. *yunnanensis* and var. *tenuifolia* are tall arborescent forms reaching up to 30 m, occurring across a wide elevational range of 600–3100 m and in riverine habitats at 400–1200 m, respectively. In contrast, var. *pygmaea* is a multi‐stemmed shrub that grows to only about 2 m and is typically found on dry, nutrient‐poor, south‐facing slopes at elevations of 2200–3100 m. Substantial intraspecific variation in serotiny also exists within *P. yunnanensis* : the arborescent varieties at lower elevations generally exhibit low levels of cone serotiny, whereas the shrubby var. *pygmaea* inhabiting dry, nutrient‐limited slopes shows markedly higher serotiny levels (Zhou et al. 2022) (Figure 1). These pronounced differences in morphology and habitat conditions provide a natural framework for investigating how fire and environmental factors shape serotiny in *P. yunnanensis* . Previous studies have observed intraspecific variation in serotiny among populations, and this study aims to identify the physiological and ecological factors, including climatic variables, soil properties, fire regime characteristics, and cone chemical traits that influence serotiny, thereby offering new insights into its adaptive evolution in this species.

## 2 Materials and Methods

### 2.1 Experimental Material Collection

**FIGURE 2:**
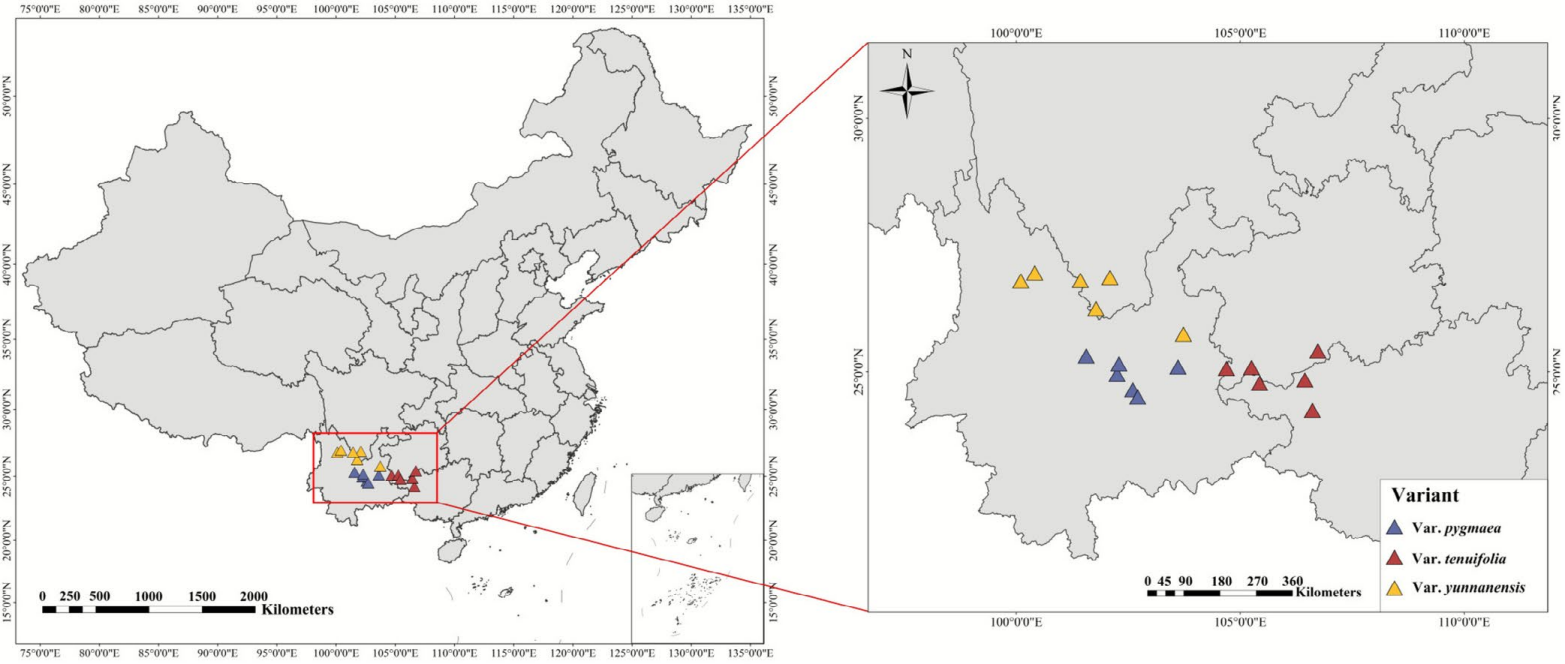
Distribution of three variants of *P. yunnanensis* populations sampled across southwestern China, as illustrated on a map of China.

Based on the natural distribution ranges of *P. yunnanensis* , field surveys and sample collections were conducted across four provinces in southwestern China: Sichuan, Yunnan, Guizhou, and Guangxi. A total of 18 *P. yunnanensis* natural populations were selected (6 for *P. yunnanensis* var. *pygmaea*, 6 for *P. yunnanensis* var. *tenuifolia*, and 6 for *P. yunnanensis* var. *yunnanensis*, Figure 2). Pollen dispersal typically takes place in April and May, while cones reach maturity in October of the subsequent year. In each sample forest stand, three 20 m × 20 m plots were established. From each plot, five well‐grown, mature, and representative individuals were selected for investigation. Because of the striking differences in growth form among the three varieties, the sampling protocol was adjusted accordingly. For *P. yunnanensis* var. *pygmaea*, a low‐growing, multi‐stemmed shrub (1–2 m tall), all cones in the entire crown were recorded. In contrast, for the tall‐tree varieties ( *P. yunnanensis* var. *yunnanensis* and var. *tenuifolia*, 15–30 m tall), cones were collected from three healthy and disease‐free branches located in the upper, middle, and lower crown positions following the method of (Tapias et al. 2001; Hernández‐Serrano et al. 2013). The adjustment in serotiny level investigation was made because, for tall‐tree varieties, it is impractical to count every cone due to the great height and dense branches. To validate this approach, we performed a comparison on trees where a full count was possible—specifically, shorter individuals or those with less dense foliage. We found no significant difference between the full‐tree count and our method of sampling three representative branches. For soil sampling, five points were selected in each plot using the five‐point sampling method (Liu et al. 2021; Li et al. 2022). Soil samples were collected from the 0–20 cm layer, labeled, packaged, and transported to the laboratory for further analysis (Li et al. 2021; Zhou et al. 2025).

### 2.2 Traits Measurement

#### 2.2.1 Tree Height and Serotiny Level

At each sampling site, six mature individuals were selected for tree height measurement. A laser rangefinder, leveling instrument, and leveling rod were used in combination to obtain accurate values. First, a horizontal reference line was established using the leveling instrument and rod. The laser rangefinder was positioned along this line and kept stable throughout the measurement process. To determine tree height, the laser beam was first aimed at the base of the tree to record the horizontal distance (L_1_) and the angle (α_1_) between the beam and the horizontal plane. The beam was then directed toward the top of the tree to obtain the slant distance (L_2_) and the angle (α_2_). The vertical offset (L_0_) between the rangefinder and the tree base was also recorded. Tree height (in meters) was calculated using the following formula:

$$\text{Tree height}\;\left(m\right)=L_{0}\times \tan a_{2}+L_{1}\times \sin a_{1}$$

Pollination in *Pinus yunnanensis* occurs from late March to early April. After fertilization, the young cones acquire their characteristic shape within the same year but do not increase further in size, whereas unfertilized cones gradually wither during that period. In the following spring (late March), as new shoots begin to elongate, the fertilized cones resume growth, enlarging over the next 7–8 months and reaching full maturity by December. This clear, sequential, and rhythmically regulated developmental process reflects the species' characteristic biennial reproductive cycle. Each year, only one whorl of new shoots is produced at the distal end of the main stem and lateral branches, forming a distinct annual ring at the junction between new and older growth, where cones are borne. Consequently, the position of cones along the annual branch nodes provides a reliable basis for determining the year of cone formation and the duration of serotiny, without requiring multi‐year field observations (Jin and Peng 2004; Su et al. 2015, 2019). To assess the level of serotiny, the number of cones that had matured in their second year of development (Q_1_) and the number of cones that remained closed and attached to the branches after ripening (Q_2_) were recorded. So the serotiny level was calculated as (Goubitz et al. 2004):

$$\text{Serotiny level}=\frac{Q2}{Q1}\times 100\%$$

#### 2.2.2 Resin Content Determination

The resin content of cones was determined using the Soxhlet extraction method (Luque de Castro and Priego‐Capote 2010; Simões et al. 2024). Samples were placed in porous thimbles and continuously washed with hexane, which was regenerated through distillation and siphoning. This process effectively removed the analyte, after which the solvent was evaporated, leaving behind the extracted resin. The resin was then weighed for quantification.

#### 2.2.3 Cellulose Content Analysis

Cellulose content was measured via the phenol‐sulfuric acid method using a UV–Vis spectrophotometer that relies on a colorimetric reaction, with absorbance values used to calculate cellulose concentration (Cazón et al. 2022; Huang and Yu 2019).

#### 2.2.4 Ash Content Measurement

Ash content of cone scales was determined by incinerating plant samples in a muffle furnace at 600°C, which ensured complete combustion of organic matter, with the remaining inorganic residue weighed to determine ash content (Ismail 2017; Li et al. 2023; Zeng et al. 2014).

#### 2.2.5 Soil Sample Analysis

Soil samples were air‐dried and sieved (100‐mesh) before analysis. Soil pH was measured using a calibrated pH meter (Schofield and Taylor 1955; Kissel et al. 2012). Organic carbon content was determined via potassium dichromate oxidation (Shaw 1959; Bremner and Jenkinson 1960), total nitrogen was quantified using the Micro‐Kjeldahl method (Bremner 1960; Aguirre 2023), total phosphorus was assessed by the Molybdenum‐Antimony Colorimetric method (Tandon et al. 1968; Dick and Tabatabai 1977), and total potassium was analyzed via flame photometry (Kolterman and Truog 1953).

### 2.3 Environmental Data Collection

Nineteen bioclimatic variables and latitude data were obtained from the World Climate Database (WorldClim: https://www.worldclim.org/data/index.html). We used the high spatial resolution (30 arc‐s, ~1 km at the Equator) from this database (Table 1) (Hijmans et al. 2005). The 30 arc‐second resolution means that the data cover the globe in a grid of about 1 km, with each grid point providing an average value of a climate variable.

**TABLE 1:** 19 bioclimatic variables were extracted from WorldClim (https://www.worldclim.org/data/index.html); these environment variables use the 30 arc‐s version of the study.

Environmental variables	Description	Unit
Bio1	Annual Mean Temperature	°C
Bio2	Mean Diurnal Range	°C
Bio3	Isothermality	
Bio4	Temperature Seasonality	
Bio5	Max Temperature of Warmest Month	°C
Bio6	Min Temperature of Coldest Month	°C
Bio7	Temperature Annual Range	°C
Bio8	Mean Temperature of Wettest Quarter	°C
Bio9	Mean Temperature of Driest Quarter	°C
Bio10	Mean Temperature of Warmest Quarter	°C
Bio11	Mean Temperature of Coldest Quarter	°C
Bio12	Annual Precipitation	mm
Bio13	Precipitation of Wettest Month	mm
Bio14	Precipitation of Driest Month	mm
Bio15	Precipitation Seasonality	mm
Bio16	Precipitation of Wettest Quarter	mm
Bio17	Precipitation of Driest Quarter	mm
Bio18	Precipitation of Warmest Quarter	mm
Bio19	Precipitation of Coldest Quarter	mm

Wildfire regime characteristics are important environmental factors influencing plant traits. However, accurately characterizing fire regimes of a given region remains a persistent challenge, particularly in the absence of long‐term historical records or detailed empirical data. In this study, we used active fire data (2001–2024) from the Fire Information for Resource Management System (FIRMS) to characterize the fire regime at each sampling site. To ensure consistent data availability across all sampling locations, we aggregated the original 1 km spatial resolution data to a 0.5° × 0.5° grid (approximately 55 km^2^), as many sites contained no wildfire detection at the finer resolution. Within each aggregated grid cell, wildfire activity was quantified using two metrics: Fire Activity (FA)—the total number of fire detection points and Fire Radiative Power (FRP)—the mean FRP value of all detected fires within the grid cell.

### 2.4 Statistical Analyses

Variation in serotiny level among *Pinus yunnanensis* populations was analyzed using one‐way ANOVA. Before conducting multiple regression analysis, we examined multicollinearity among environmental variables to minimize autocorrelation effects. Based on correlation analyses and ecological relevance, we retained a set of representative variables related to climate, soil, fire regime, and cone chemistry. Serotiny level was used as the dependent variable in the regression model (Groemping 2007; Lai et al. 2022). Subsequently, to further explore the drivers of changes in serotiny level during stand development, we used structural equation modeling (SEM) via the ‘piecewise’ package in R 4.4.1 to examine the interactions between serotiny level and environmental factors. SEM enables the investigation of relationships between latent and observed variables, as well as between latent variables, through a combined measurement and structural model (Bollen 1989; Lefcheck 2016; Davvetas et al. 2020) represented by the following equations:

X=Λxξ+δ

Y=Λyη+ε

η=Bη+Гξ+ζ

X and Y denote vectors of observed exogenous and endogenous variables, respectively. *η* represents the vector of latent endogenous variables, while ξ denotes the vector of latent exogenous variables. Λx and Λy are the factor loading matrices of X on ξ and Y on *η*, respectively. B is the path coefficient matrix among latent endogenous variables, and Γ represents the path coefficient matrix from latent exogenous to latent endogenous variables. δ and ε are the error terms associated with the observed exogenous and endogenous variables, respectively, and ζ is the residual term in the structural equation. Model fit was evaluated using the Akaike Information Criterion (AIC) and Fisher's C statistic, with a non‐significant C test (*p* > 0.05) indicating a good fit between the model and the data (Akaike 1974, 1978; Grace 2006). All statistical analyses and graphical visualizations were conducted in R 4.4.1. Unless otherwise specified, statistical significance was determined at the *p* < 0.05 level.

## 3 Results

### 3.1 The Variation in Serotiny Level Within *Pinus Yunnanensis*

**FIGURE 3:**
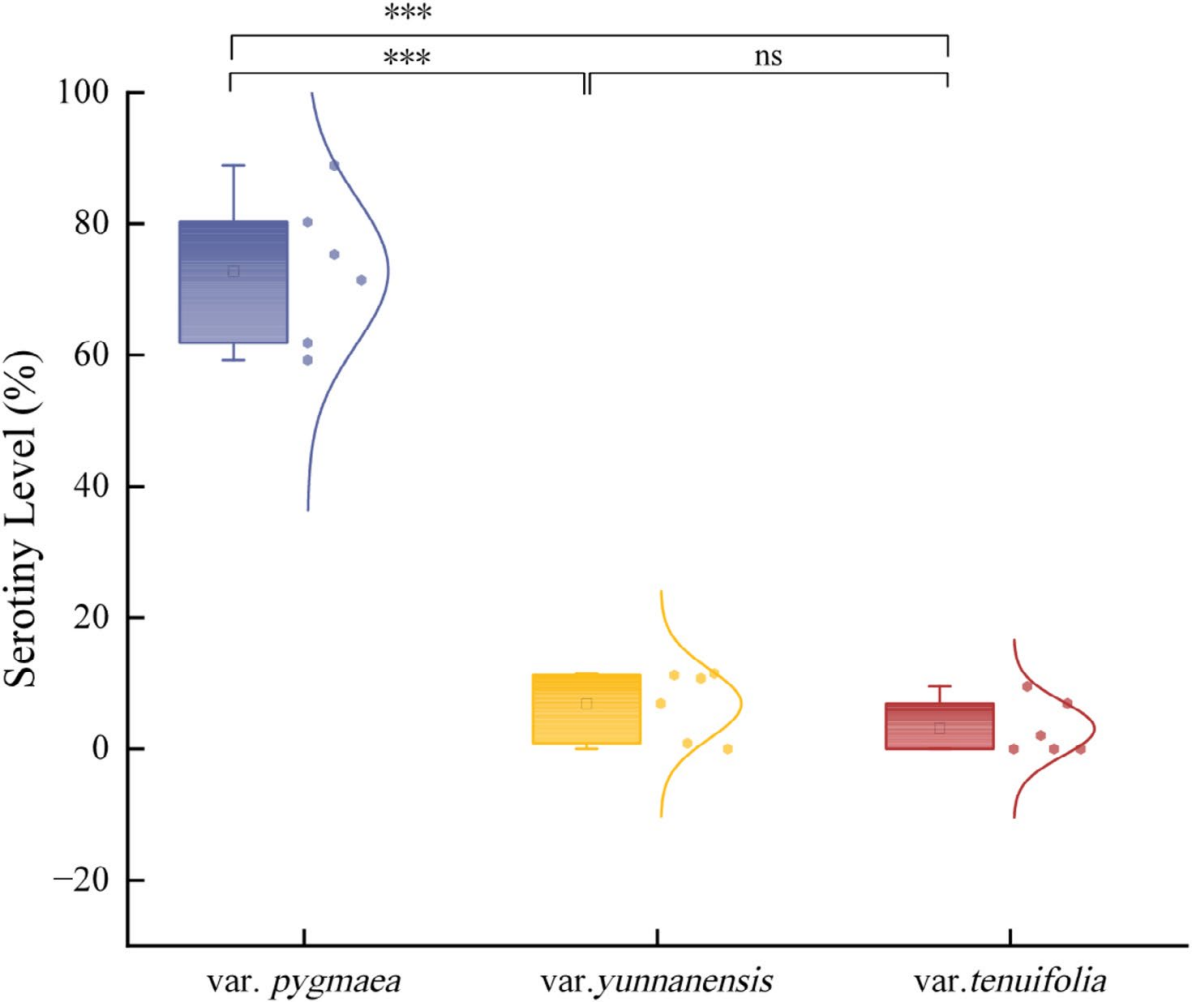
Comparison of the serotiny level of pinecones of three varieties of *Pinus yunnanensis* (mean ± standard deviation). “***” indicates highly significant differences (*p* < 0.001), and “ns” indicates no significant differences (*p* > 0.05).

The serotiny level varied significantly among the three varieties of *Pinus yunnanensis*. In var. *pygmaea*, serotiny level ranges from 59.26% to 88.89%, with a mean of 72.17%. In contrast, var. *yunnanensis* exhibited much lower serotiny level, ranging from 0.02% to 11.50% (mean = 6.55%), while var. *tenuifolia* showed similarly low values, ranging from 0.00% to 9.54% (mean = 3.26%) (Table 2). The highest recorded serotiny level was observed in the Qinfeng population of var. *pygmaea* (88.89%), whereas complete non‐serotiny (0.00%) was found in three populations of var. *tenuifolia* (Wusha, Lingyun, and Luodian). Statistical analysis showed that serotiny level in var. *pygmaea* was significantly higher than those in the other two varieties (*p* < 0.001), while no significant difference was detected between var. *yunnanensis* and *var. tenuifolia* (Figure 3).

**TABLE 2:** Serotiny level of three varieties (var. *pygmaea*, var. *yunnanensis*, and var. *tenuifolia*) of *Pinus yunnanensis* across 18 sample plots.

Variant	Sample plot	Altitude (m)	Longitude	Latitude	Serotiny level (%)
*Pinus yunnanensis* var. *pygmaea*	Mouding	1967	101.56	25.32	61.85
	Jinning	1895	102.60	24.64	59.26
	Luliang	1968	103.61	25.10	75.30
	Lubiao	2314	102.25	24.95	80.29
	Qinfeng	2205	102.29	25.16	88.89
	Liujie	2066	102.71	24.49	71.40
*Pinus yunnanensis* var. *yunnanensis*	Datian	1671	101.78	26.27	6.93
	Yaoan	1555	100.10	26.82	11.26
	Gucheng	2116	100.41	26.98	0.85
	Miyi	1295	102.09	26.89	10.75
	Huimin	1399	101.43	26.83	11.50
	Shuichong	1931	103.73	25.76	0.02
*Pinus yunnanensis* var. *tenuifolia*	Wusha	613	104.69	25.07	0.00
	Xinqiao	751	105.25	25.09	9.54
	Longlin	703	105.43	24.77	2.08
	Lingyun	504	106.61	24.22	0.00
	Leye	930	106.44	24.84	6.94
	Luodian	377	106.73	25.42	0.00

### 3.2 Factors Affecting the Serotiny Level in *Pinus Yunnanensis*

**FIGURE 4:**
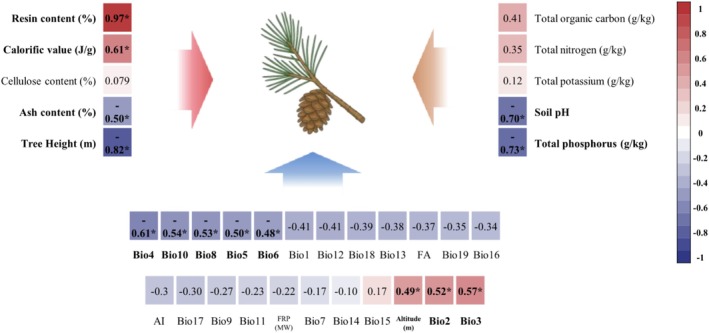
Pearson correlation matrix between serotiny level and all traits. The color gradient represents the strength and direction of correlation coefficients (red: Positive correlation; blue: Negative correlation). Asterisks indicate levels of statistical significance (*p < 0.05).

Serotiny level was positively associated with resin content (*p* < 0.001) and calorific value (*p* < 0.05) of cone scales. In contrast, a significant negative correlation was observed between serotiny and tree height (*p* < 0.05). Among the climatic variables, serotiny level showed a positive correlation with Bio2 and Bio3 (*p* < 0.05); conversely, negative correlations were observed with Bio4, Bio5, Bio6, Bio8, and Bio10 (*p* < 0.05). Regarding soil physicochemical properties, total phosphorus content exhibited a strong negative correlation with serotiny (*p* < 0.001), while soil pH was also negatively correlated (*p* < 0.01). In contrast, no significant correlations were found between serotiny and total organic carbon, total nitrogen, or total potassium (Figure 4).

### 3.3 Drivers and Pathways Influencing Serotiny in *Pinus Yunnanensis*

**FIGURE 5:**
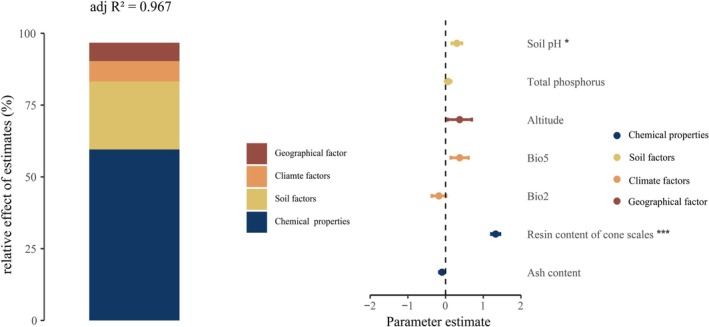
The relative contributions of multiple predictor variables to the serotiny level in *Pinus yunnanensis* (expressed as percentages of explained variance), along with the mean parameter estimates (standardized regression coefficients) and their 95% confidence intervals for each predictor included in the model.

Totally, 13 factors were identified as significantly correlated with serotiny level in *P. yunnanensis* . To avoid multicollinearity, highly autocorrelated variables were excluded from subsequent analyses. Eight variables were retained and then included in a multiple linear regression model to evaluate their relative contributions to the variation in serotiny level. In the optimal predictive model, soil pH and cone resin content emerged as significant predictors (Figure 5). Variance partitioning revealed that cone chemical traits, soil properties, climatic factors, and geographic location explained 61.58%, 24.53%, 7.31%, and 6.60% of the total variation in serotiny, respectively, with the full model accounting for 96.7% (Table 3 and Figure 5).

**TABLE 3:** Proportional contribution of grouped predictor variables (cone chemical traits, soil properties, climatic factors, and geographic location) to the total variance in serotiny level, as determined by the multiple regression model.

Environmental factors	Category	*R* ^2^	Contribution (%)
Altitude (m)	Geographical factor	0.063	6.60
Bio2 (°C)	Climate factors	0.070	7.31
Bio5 (°C)			
Soil pH	Soil factors	0.237	24.53
Total phosphorus (g/kg)			
Resin content of cone scales (%)	Chemical properties of cones	0.596	61.58
Ash content (%)			

**FIGURE 6:**
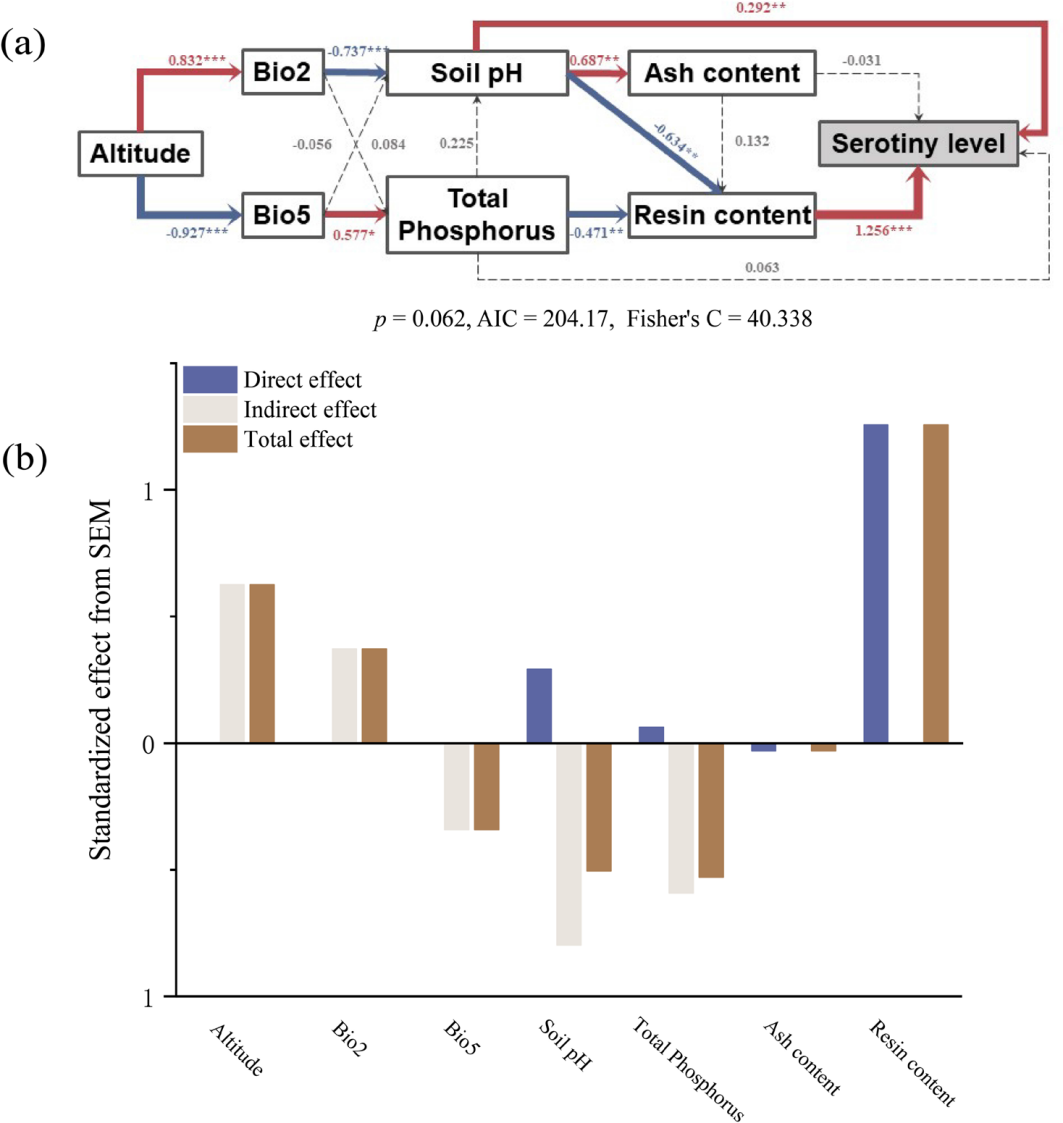
(a) A piecewise structural equation model (SEM) was used to examine direct and indirect relationships among variables, with path coefficients indicating the strength of these effects (b) and levels of significance (***p < 0.001; **p < 0.01; *p < 0.05, and unmarked indicates *p* > 0.05). The numerical values adjacent to the arrows represent load factors between parameters. Solid blue and red arrows denote positive and negative relationships, respectively, with arrow width corresponding to the relationship strength. Dashed lines indicate non‐significant relationships (*p* > 0.05). Serotiny level correlations with their associated variables are shown using blue upward (↑, positive) and red downward (↓, negative) arrows.

These variables were further used to construct the structural equation model and to assess whether they exert direct or indirect effects on serotiny level. Results from the piecewise structural equation modeling revealed that altitude positively influenced Bio2 and negatively influenced Bio5, which affected soil pH and total phosphorus content. Specifically, Bio2 indirectly influenced resin content by reducing soil pH, thereby exerting a positive regulatory effect on serotiny level. Soil pH itself showed a significant correlation with serotiny. In contrast, Bio5 negatively impacted resin content by increasing total phosphorus levels, which in turn indirectly suppressed serotiny. Regarding cone chemical traits, both soil pH and total phosphorus significantly reduced serotiny by decreasing resin content. Although ash content showed a slight negative association with serotiny, the effect was weak (path coefficient = −0.031). Resin content had the strongest positive effect on serotiny (path coefficient = 1.256, *p* < 0.001). Total effect analysis further indicated that resin content had the greatest influence on serotiny (total effect = 1.256), followed by altitude (0.626) and Bio2 (0.372), while Bio5 (−0.341), soil pH (−0.504), and total phosphorus (−0.529) exhibited substantial negative effects (Figure 6).

## 4 Discussion

### 4.1 Intraspecific Variations in Serotiny Level Within *Pinus Yunnanensis*

Southwest China has a subtropical monsoon climate characterized by alternating warm‐dry and hot‐humid seasons (Guo et al. 2021; Peng et al. 2024), creating conditions conducive to frequent wildfires (Ying et al. 2018; Qiao et al. 2020; Jing et al. 2023). Consequently, many plant species in this region have developed fire‐adaptive traits to survive wildfires (Keeley et al. 2011; Keeley 2012; Su et al. 2015, 2019). Serotiny plays a critical role in the reproduction, population persistence, and ecological niche expansion of *Pinus yunnanensis*, contributing to its adaptability to a wide range of environmental conditions (Budde et al. 2014; Song et al. 2024).

*Pinus yunnanensis* var. *pygmaea* exhibits the highest serotiny level, indicating it adopts a “fire‐embracing” strategy which enhances its resilience to frequent wildfires (Pausas 2015a; Wang et al. 2024). This adaptive strategy promotes rapid population regeneration during the post‐fire period (Canelles et al. 2019; Agne et al. 2022). The high serotiny level of this variety ensures the synchronized release of seeds following fire events, thereby maximizing reproductive success (Han et al. 2015; Zhang et al. 2018, 2025). In contrast, *P. yunnanensis* var. *tenuifolia* and var. *yunnanensis*, which exhibit a lower serotiny level, adopt a “fire‐tolerant” strategy which enables these varieties to persist in frequent but low‐intensity surface fires (Tang et al. 2013; Han et al. 2015; Bai et al. 2020).

### 4.2 Factors Influencing Serotiny Level Within *P. yunnanensis*

Serotiny level is strongly influenced by the resin content of the cones. The resinous substances seal the cone scales, delaying seed release until fire or elevated temperatures weaken this barrier (Harlow et al. 1964; Wang et al. 2024). Beyond resin content, environmental factors also play critical roles in shaping serotiny level and consequently influence the reproductive strategies of *P. yunnanensis* . For instance, a greater diurnal temperature range (Bio2) indicates substantial fluctuations in daily temperature, which can impose environmental stress on plants and influence the timing of cone development and seed release. Such variability has been linked to conservative reproductive strategies, including delayed seed release that enhance offspring survival in unpredictable environments (Moya et al. 2008). Regions characterized by large diurnal temperature amplitudes, often at higher elevations or in semi‐arid zones, also tend to experience more variable fire regimes (Larson et al. 2009; Archibald et al. 2013), potentially selecting for traits that maintain cone closure through increased resin accumulation (Tapias et al. 2001; Clarke et al. 2013). In contrast, extremely high maximum temperatures during the warmest month (Bio5) may compromise cone integrity and resin stability, leading to premature opening or reduced resin production. Elevated thermal stress can alter the biosynthesis and viscosity of terpenoid resins (Knapp and Anderson 1980). Thereby weakening the serotinous mechanism. Thus, while moderate temperature variability may promote serotiny as an adaptive response to environmental uncertainty, sustained high temperature extremes can suppress it by destabilizing cone structure and resin sealing capacity (Madrigal et al. 2021; Lopez et al. 2025).

Soil properties also play a critical role in regulating serotiny level, acting through both physiological constraints and ecological trade‐offs. Soil pH and total phosphorus content were found to be negatively correlated with serotiny level, consistent with findings that nutrient‐poor or acidic soils often favor stronger serotiny (Lamont 1991; Enright et al. 1998b; Orians and Milewski 2007). In such environments, plants tend to adopt conservative life‐history strategies, prioritizing long‐term reproductive assurance over immediate seed dispersal. Previous studies have shown that alkaline soils can reduce the bioavailability of key micronutrients, even under otherwise nutrient‐rich conditions (Rengel 2015). This limitation may restrict nutrient uptake and photosynthetic efficiency, thereby influencing carbon allocation patterns within the plant. Since the maintenance of closed cones and the synthesis of protective resins are energetically costly, trees under such physiological constraints may reduce investment in serotiny to prioritize survival and vegetative growth. Similarly, high phosphorus availability has been linked to greater vegetative vigor and continuous seedling recruitment, reducing dependence on fire‐cued mass germination strategies (Larson and Funk 2016; Tang et al. 2021). Consequently, the evolutionary advantage of serotiny may diminish in phosphorus‐rich habitats where fire occurrence is infrequent or variable (Certini 2005; Huot et al. 2014; Fuentes‐Ramirez et al. 2022). Overall, these results suggest that serotiny level in *P. yunnanensis* reflects a complex interaction between edaphic constraints and reproductive ecology, in which non‐fire environmental factors exert a significant and multifaceted influence. This highlights the possibility that serotiny in *P. yunnanensis* is more strongly influenced by non‐fire environmental variables, such as temperature, soil chemistry, and resource allocation, rather than by the fire regime alone (Tapias et al. 2004; Keeley et al. 2011; Mays et al. 2017).

Structural equation modeling (SEM) reveals that the serotiny level of *Pinus yunnanensis* is shaped not only by fire‐related factors but also by a network of direct and indirect effects stemming from climate, topography, soil properties, and cone traits. The model highlights that elevation indirectly promotes serotiny by regulating key bioclimatic variables (Bio2 and Bio5), which in turn affect soil chemistry and cone resin composition. Specifically, a greater mean diurnal temperature range (Bio2) positively influences serotiny by reducing soil pH, thereby enhancing resin content accumulation: a key determinant of cone closure. Such a relationship is likely to arise because strong daily temperature fluctuations accelerate mineral weathering and organic matter decomposition, thereby releasing acidic ions (Kuzyakov and Blagodatskaya 2015; Fang et al. 2019). Acidic soils tend to limit nutrient availability and stimulate the synthesis of carbon‐based defensive compounds (Fageria and Baligar 2008; Zaman et al. 2025), including resins, reinforcing serotiny as a stress‐tolerance strategy (Herms and Mattson 1992; Pausas et al. 2004, 2021). In contrast, a higher maximum temperature during the warmest month (Bio5) was associated with increased soil phosphorus availability, which in turn negatively affected resin synthesis and ultimately reduced serotiny. Elevated temperatures accelerate the mineralization of organic matter and the weathering of parent materials, thereby releasing phosphorus into the soil solution (Sarria‐Villa et al. 2021; Moura et al. 2025). Higher P availability may alleviate nutrient limitation and shift carbon allocation from the production of secondary metabolites, such as terpenoid resins, toward primary growth processes (Veneklaas et al. 2012; Jahan et al. 2025). Under such warm and fertile conditions, trees invest less in carbon‐based defenses, leading to reduced resin accumulation and weakened cone‐sealing capacity (Hedhly et al. 2009; Ul Hassan et al. 2022; Qian et al. 2025). Prolonged heat exposure may further impair the enzymatic and physiological mechanisms responsible for maintaining cone closure (Wyse et al. 2019; Tada et al. 2024). Moreover, both soil pH and total phosphorus content exhibited significant negative indirect effects on serotiny by suppressing resin production. Acidic, nutrient‐poor soils stimulate resin synthesis as a defensive allocation strategy, thereby enhancing serotiny, whereas high phosphorus availability promotes growth investment and reduces the reliance on delayed seed release. Among cone traits, resin content showed the strongest direct positive effect on serotiny, highlighting its crucial physiological role in maintaining cone dormancy until fire or other environmental cues trigger seed release (Clarke et al. 2013).

Collectively, these findings indicate that serotiny in *Pinus yunnanensis* is a consequence of complex environmental filtering, whereby climate and soil factors regulate resin‐based defense strategies (Aitken et al. 2008; Vázquez‐González et al. 2020). This integrated pathway perspective offers novel insights into the evolution of plant traits under multifactorial environmental pressures (Bellard et al. 2012; Pacifici et al. 2017), thereby informing predictions of trait responses to climate change. Our findings highlight the interactive effects of climate and soil on the serotiny level of *P. yunnanensis* . Structural equation modeling (SEM) confirms that environmental conditions regulate resource allocation strategies (Kerkhoff et al. 2006; Funk and Vitousek 2007; Reich et al. 2008), with temperature and soil fertility modulating the physiological mechanisms underpinning serotiny. This integrated approach provides a framework for predicting how future climate shifts may shape the adaptive traits and distribution of fire‐adapted species (Niinemets 2001; Wright et al. 2004).

### 4.3 The Effects of Fire Regime Characteristics on Serotiny Across *Pinus Yunnanensis*

Fire is widely recognized as a major ecological force driving the evolution of serotiny in many pine species (Bond and Keeley 2005; He et al. 2011, 2019; Pausas 2015b). However, our results did not detect significant differences in fire occurrence or fire radiative power we used in this study among populations of *Pinus yunnanensis*. We argue that this null result does not preclude a significant role for fire in the evolution of serotiny. A likely explanation is that the two satellite‐derived fire metrics used in this study may not adequately capture the nuanced characteristics of the local fire regimes. While satellite data provide broad‐scale information, it can fail to resolve critical fire attributes, such as intensity, severity, fire type, and seasonality, which are more relevant to evolutionary processes.

Accurately characterizing fire regimes remains a persistent challenge, particularly in the absence of long‐term historical records or detailed empirical data from a given location. Our use of this data was therefore an initial attempt to detect fire regime variation across populations exhibiting trait divergence. Future research could benefit from integrating higher‐resolution and long‐term fire data to capture critical fire attributes such as intensity, severity, seasonality, and crown and surface fires. Combining historical fire reconstructions, dendrochronology, and controlled experiments would allow a more precise evaluation of how fire interacts with climatic and edaphic factors to shape serotiny. Moreover, exploring the combined effects of fire, soil, and climate using mechanistic or predictive models could provide deeper insights into the evolutionary and ecological dynamics of serotiny across *Pinus yunnanensis* populations.

Despite these advances, determining which metrics best quantify fire regimes and how to meaningfully incorporate them into ecological analyses remains an open challenge. Fire regimes are inherently multidimensional and cannot be fully captured by any single indicator or data source. Identifying metrics that reflect the ecological processes most relevant to serotiny will require further methodological innovation and empirical testing. In addition, fire and vegetation influence one another through reciprocal feedback: vegetation structure shapes fire behavior, while repeated fires modify plant traits and population strategies over time. Recognizing this bidirectional interaction is essential for future work aimed at disentangling the mechanisms through which fire shapes serotiny in *Pinus*.

## 5 Conclusion

Our research revealed significant intraspecific variation in serotiny levels among the three varieties of *Pinus yunnanensis*. Environmental factors: temperature, precipitation variability, soil pH, and total phosphorus content significantly influence serotiny expression in this species. Structural equation modeling demonstrated that serotiny is primarily driven by resin content, which is indirectly regulated by altitude, climatic variables (Bio2 and Bio5), and soil properties (pH and total phosphorus). These findings provide new insights into the adaptive evolution of serotiny in *P. yunnanensis* and underscore the complex interplay between environmental factors and fire‐related traits in fire‐prone ecosystems.

## Author Contributions

**Ying Liu:** formal analysis (equal), investigation (equal), software (equal), visualization (equal), writing – original draft (equal). **Haichuan Lin:** formal analysis (equal), investigation (equal), software (equal), visualization (equal). **Dongli Yu:** formal analysis (equal), investigation (equal), software (equal). **Zihan Zhang:** investigation (equal), software (equal), visualization (equal). **Wuchao Gao:** formal analysis (equal), investigation (equal), software (equal). **Dongyu Cao:** data curation (equal), resources (equal). **Dachuan Dai:** data curation (equal), investigation (equal), resources (equal). **Xinglei Cui:** conceptualization (equal), conceptualization (equal), funding acquisition (equal), funding acquisition (equal), methodology (equal), methodology (equal), project administration (equal), resources (equal), resources (equal), writing – review and editing (equal), writing – review and editing (equal).

## Funding

This study is supported by the National Natural Science Foundation of China (grant numbers 32422060, 32101532).

## Conflicts of Interest

The authors declare no conflicts of interest.

## Supporting information


**Data S1:** ece372838‐sup‐0001‐DataS1.zip.

## Data Availability

The data supporting the findings of this study are publicly available in the Dryad Digital Repository via a permanent DOI: https://doi.org/10.5061/dryad.8gtht771d.
